# High-quality reference genome and annotation aids understanding of berry development for evergreen blueberry (*Vaccinium darrowii*)

**DOI:** 10.1038/s41438-021-00641-9

**Published:** 2021-11-01

**Authors:** Jiali Yu, Amanda M. Hulse-Kemp, Ebrahiem Babiker, Margaret Staton

**Affiliations:** 1 Genome Science and Technology Program, University of Tennessee, Knoxville, TN 37996 USA; 2 USDA-ARS Genomics and Bioinformatics Research Unit, Raleigh, NC USA; 3 Department of Crop and Soil Sciences, North Carolina State University, Raleigh, NC USA; 4 USDA-ARS Thad Cochran Southern Horticultural Laboratory, Poplarville, MS USA; 5 Department of Entomology and Plant Pathology, University of Tennessee, Knoxville, TN 37996 USA

**Keywords:** Genomic analysis, Genome, Transcriptomics, Non-coding RNAs

## Abstract

*Vaccinium darrowii* Camp (2*n* = 2*x* = 24) is a native North American blueberry species and an important source of traits such as low chill requirement in commercial southern highbush blueberry breeding (*Vaccinium corymbosum*, 2*n* = 4*x* = 48). We present a chromosomal-scale genome of *V. darrowii* generated by the combination of PacBio sequencing and high throughput chromatin conformation capture (Hi–C) scaffolding technologies, yielding a total length of 1.06 Gigabases (Gb). Over 97.8% of the genome sequences are scaffolded into 24 chromosomes representing the two haplotypes. The primary haplotype assembly of *V. darrowii* contains 34,809 protein-coding genes. Comparison to a *V. corymbosum* haplotype assembly reveals high collinearity between the two genomes with small intrachromosomal rearrangements in eight chromosome pairs. With small RNA sequencing, the annotation was further expanded to include more than 200,000 small RNA loci and 638 microRNAs expressed in berry tissues. Transcriptome analysis across fruit development stages indicates that genes involved in photosynthesis are downregulated, while genes involved in flavonoid and anthocyanin biosynthesis are significantly increased at the late stage of berry ripening. A high-quality reference genome and accompanying annotation of *V. darrowii* is a significant new resource for assessing the evergreen blueberry contribution to the breeding of southern highbush blueberries.

## Introduction

Blueberries belong to the genus *Vaccinium* L. section Cyanococcus which contains species at the diploid, tetraploid and hexaploid levels^1^. Although the genus *Vaccinium* L. contains about 450 species, only three groups, lowbush (*Vaccinium angustifolium*, 2*n* = 4*x* = 48), highbush (*Vaccinium corymbosum* L. 2*n* = 4*x* = 48), and rabbiteye (*Vaccinium virgatum*, 2*n* = 6*x* = 72), are cultivated^2^. Since the establishment of blueberry breeding programs, desirable traits from native *Vaccinium* species have been bred into cultivated forms to extend the range of blueberry production, to tolerate abiotic stress such as heat and drought, and to increase disease resistance^3–5^. The recent expansion of highbush blueberry production into the southern states is due to the development of low chill southern highbush blueberry (SHB) cultivars from crosses between the northern highbush and native *Vaccinium* species from the southeastern United States^6^. Three species, *Vaccinium darrowii* Camp, *Vaccinium myrsinites* Lam., and *V. virgatum* were extensively used in SHB cultivars development^7^.

*V. darrowii* Camp. is an evergreen wild blueberry characterized by low chilling requirement, small leathery and thick leaves, short-stature, and twiggy growth^8^. *V. darrowii* clone Florida 4B was used extensively in breeding programs to reduce the chilling requirement of northern highbush blueberries. One striking feature of the *V. darrowii* clone Florida 4B is the production of unreduced gametes which allowed it to act as a “bridging species” to overcome sterility barriers between polyploid and several diploid *Vaccinium* species^9^. There is a continual need to broaden the genetic base of SHB cultivars and enrich them with alleles from other species to enhance fruit quality and improve the level of tolerance to biotic and abiotic stresses^4^. *V. darrowii* continues to be an important source of traits; SHB cultivars lack resistance to the blueberry leaf rust pathogen *Thekopsora minima* while *V. darrowii* exhibits a high level of resistance^10^. Growing interest to continue to utilize diverse *V. darrowii* accessions for breeding could be bolstered by new genomic resources^11,12^.

Although *V. darrowii* has a low chilling requirement, it has a longer fruit development period than southern highbush *V. corymbosum*^13^. Small berries, late-ripening, and dwarf growth habitats make *V. darrowii* unsuitable for commercial production. Previous research has largely focused on the development of blueberries from cultivated varieties, missing an opportunity to understand both the similarities and differences among other species used in breeding programs. Blueberries are rich in antioxidants, with increased anthocyanins and decreased flavonols during berry ripening^14^. Colle et al.^15^ reported that antioxidant contents in blueberries were in the highest level at the green berry stage and decreased during fruit development, consistent with the findings from other berries^16^. The decrease of phenolic contents may be associated with the increasing levels of anthocyanins. Biosynthetic genes related to anthocyanins production were highly expressed in ripe berries^15^. Another interesting aspect to berry development is the production of cyanogenic glycoside as a resistance compound that is toxic to herbivores^17^. Blueberry genes involved in cyanogenic glycoside catabolic pathways were found to be highly expressed in the green berry stage of SHB and are hypothesized to prevent unripe berries from being eaten^18^. While its excellent flavor has been reported, many fruit quality and development attributes of *V. darrowii* have not been investigated.

Classical breeding approaches have been used to develop high-quality SHB cultivars. However, due to self-incompatibility, heterozygosity, and the complexity of its tetraploid genome, genomic resources that could hasten the breeding cycle are still limited. The development of a high-quality reference genome for species used in the breeding of SHB and incorporation of marker-assisted selection would allow breeders to expedite the breeding process and increase the precision and efficiency of cultivar development. Until recently, few genomic resources have been available for blueberry. The genome of the tetraploid northern highbush blueberry *V. corymbosum* ‘Draper,’ has been assembled^15^. Although this reference assembly represents the tetraploid *V. corymbosum* genome, the contribution of *V. darrowii* to the SHB gene pool remains to be clarified^19^. Comparative genomics enabled by genome sequences is a powerful tool to unravel the relationship between genomes by describing conserved regions between species within the *Vaccinium* genus. It can also be used to gain information about orthologous gene functions and structural rearrangements. Using deep-coverage Illumina sequencing in combination with PacBio and Dovetail Hi–C, the *V. darrowii* genome was assembled into twenty-four chromosome-scale scaffolds containing a 1.06 Gbp sequence.

## Results

### Haplotype representation of *V. darrowii* genome

To obtain a reference genome for *V. darrowii*, we performed whole-genome sequencing by PacBio with long-read Single-Molecule Real-Time sequencing (PacBio, CA) with 64× coverage and Illumina short-read sequencing (San Diego, CA) with 41.9× coverage. The diploid genome was assembled into 2284 contigs with a total length of 1,063,398,426 bases (Supplementary Table 1). The size of the diploid assembly was close to the estimated genome size range as measured by flow cytometry, a mean 2C-value of 1.09 ± 0.02 pg^1^. Benchmarking Universal Single-Copy Orthologs (BUSCO) analysis on the diploid genome identified 1273 out of 1614 gene orthologs (78.9%) as duplicated in the assembly (Supplementary Table 1). The diploid assembly was processed by purge_dups^20^ into a primary assembly of the most contiguous sequences representing a haploid version of the species genome. The removed contigs were kept as a secondary assembly representing the alternate haplotype. The primary assembly was scaffolded by the HiRise pipeline (Dovetail, CA) using 68.67 Gb of pair-end Hi–C sequencing data, resulting in 107 scaffolds spanning 582.7 Mb (Table 1). Over 97% of the genome length (565.7 Mb) is contained in 12 large scaffolds representing the expected 12 chromosomes. The secondary assembly was also scaffolded using the same Hi–C data^21,22^. Out of 480 Mb, 471 Mb were anchored into 12 chromosomes for the alternate haplotype. The primary and secondary assemblies were assessed by BUSCO, indicating 96.8% completeness of the 1614 gene orthologs in the primary assembly and less than 10% of those orthologs found duplicated within either assembly (Table 1). With an adjusted long terminal repeat (LTR) assembly index (LAI) score of 18.18, the primary assembly reached a ‘reference’ quality^23^, with a continuity slightly higher than the currently available ‘Draper’ genome^15^.

**Table 1:** Statistics for primary and secondary assemblies

	Primary assembly	Secondary assembly
Total length (base pairs)	582,669,857	480,504,358
Number of contigs	107	384
Chromosome length^a^	565,652,737 (97.07%)	471,549,424 (98.14%)
Contig N50	8,387,628	292,523
Scaffold N50	47,393,601	39,882,069
Number of Ns	90,150	825,887
Repetitive length^a^	266,514,449 (45.74%)	226,220,961(47.08%)
BUSCO complete (C)^b^	1,563 (96.8%) [S: 1,482 (91.8%), D: 81 (5.0%)]	1,436 (88.9%) [S: 1,311 (81.2%), D: 125 (7.7%)]
BUSCO fragmented (F)^b^	18 (1.1%)	32 (2.0%)
BUSCO missing (M)^b^	33 (2.1%)	146 (9.1%)
Annotated Genes	34,809	29,717

^a^Percentages are calculated out of bases of the genome integrated into 12 chromosomes

^b^BUSCO scores include orthologs found as complete genes in the assembly (C), which are further split into complete and single copy (S) or complete and duplicated (D). Remaining orthologs were classified as fragmented (F) or missing (M)

**Fig. 1:**
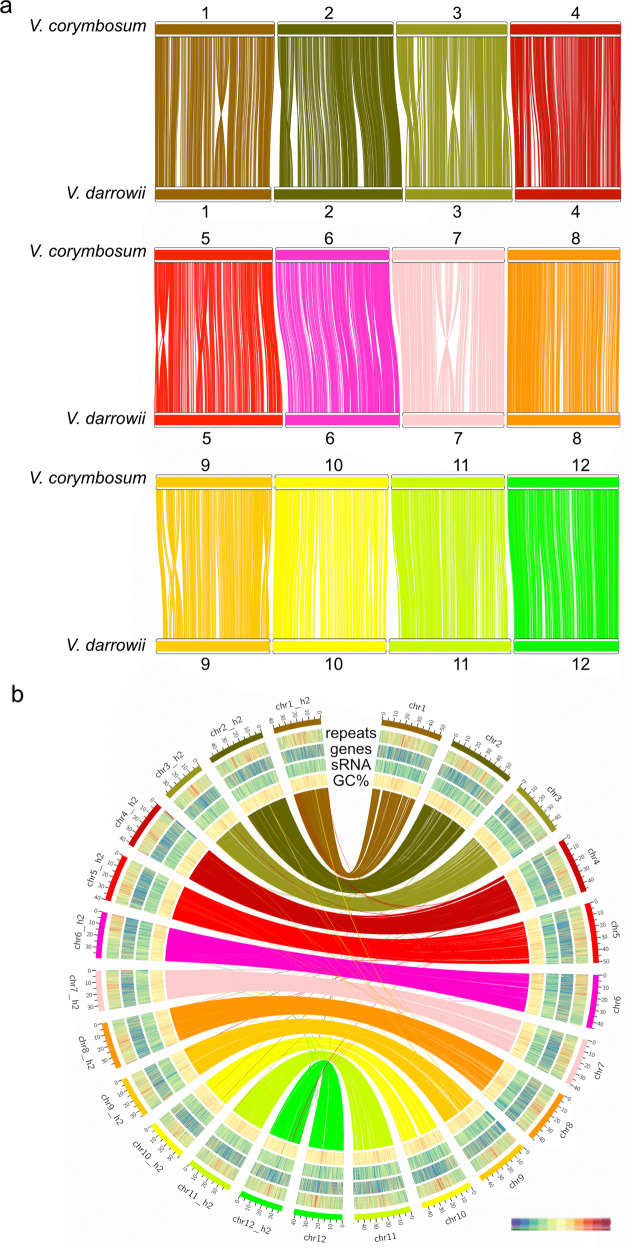
Synteny analysis of *V. corymbosum* and *V. darrowii* genome and the two haplotype assemblies of *V. darrowii*. **a** Synteny analysis between *V. corymbosum* and *V. darrowii*. **b** The genome landscape of *V. darrowii* primary and secondary assemblies. Tracks from outside to inside: repetitive element density, gene density, small RNA density, and GC content (percentage). Inner lines between chromosomes indicate synteny links between the haplotypes. The primary assembly is named chr1–12; the secondary assembly is named chr1_h2 to chr12_h2

We then compared the structure of the *V. darrowii* primary assembly to the longest single haplotype assembly (12 chromosomes) of the tetraploid *V. corymbosum* ‘Draper’ genome. To avoid large repetitive regions from generating many false alignments, we masked repetitive elements^24^. We observed a clear one-to-one chromosome mapping from the *V. darrowii* genome to *V. corymbosum*. Overall, *V. darrowii* is largely collinear to *V. corymbosum* with internal rearrangements between most homeologous chromosomes (Fig. 1a and Supplementary Fig. 1a): Chromosomes 1, 2, 3, 5, 6, 7, 9, and 10. We then ordered and named the *V. darrowii* chromosomes based on a syntenic relationship with the corresponding *V. corymbosum* chromosome naming from Colle et al.^15^.

Aligning the primary and secondary assemblies revealed overall high collinearity (Fig. 1b) but also numerous small inversions as well as the locations of gaps in the secondary assembly, some of which are likely due to the haplotype purging process and overall reduced length of the secondary assembly (Table 1 and Supplementary Fig. 2). To more accurately define the differences between the haplotypes, the PacBio reads were aligned to the primary assembly and used to identify structural variation. This analysis identified 363 well-supported structural variants of over 10 kb in length: 237 inversions, 123 duplications, and 3 insertions. This indicates the sequenced *V. darrowii* individual contains significant structural heterozygosity between haplotypes that could drive important phenotypic variation.

### Gene annotation

The annotations of two haplotype assemblies were performed independently using a combination of PacBio full-length transcript sequencing (Iso-seq) and Illumina RNA sequencing (RNA-Seq) from different berry developmental stages and 128,559 proteins from *V. corymbosum* ‘Draper’. A total of 47,371 gene loci and 49,718 transcripts were annotated in the primary genome assembly (Supplementary Table 2). Gene models were further refined using structural and functional prediction software pipelines, resulting in a final high confidence annotation of 34,809 protein-coding genes in the primary assembly and 29,717 genes in the secondary assembly (Table 1). BUSCO assessments of the total annotated protein-coding genes from the two haplotype assemblies identified 95.7% of gene orthologs as complete, which is slightly lower than the overall genome BUSCO completeness (97.5%) (Supplementary Table 1). Gene functions, gene ontology (GO) terms, and Kyoto Encyclopedia of Genes and Genomes (KEGG) objects were assigned by enTAP using protein databases including UniProt, eggNOG, and RefSeq for each of the predicted genes^25–27^. For the primary assembly annotation, 27,363 out of 34,809 (78.6%) genes were annotated with GO terms including 23,113 genes with biological processes, 19,091 genes with cellular components, and 22,839 genes with molecular functions. More than half of the protein-coding genes were involved in a metabolic process, and 328 genes were associated with antioxidant activity (Supplementary Fig. 3), suggesting antioxidant metabolic and antioxidant biosynthesis-related genes play important roles in blueberry, specifically in fruit development.

Repetitive regions were identified and annotated by both known plant repetitive element classes and de novo repeat discovery. In total, 266,514,449 bp (45.74% of the genome) were identified as repetitive elements and masked in the primary genome (Supplementary Table 3). Among all the classified repeats, the most abundant class was retroelements, specifically LTR elements with 6.10% of the genome composed of *Gypsy/DIRS1* elements, and 2.60% of the genome composed of *Ty1*/*Copia* elements (Supplementary Table 3). The density of repeats, genes, and GC content was calculated in 20-kb sliding windows over the 24 chromosomes. Gene density was lower in genomic regions with high repeat density. We observed higher GC content in the repeat-rich regions, which supports a large number of detected LTR retrotransposons (Fig. 1b).

Small RNA (sRNA) including microRNA and small interfering RNA play important roles in silencing gene expression directly by targeted degradation or indirectly through epigenetic mechanisms. sRNA loci in the *V. darrowii* genome were annotated using 17,086,934 small RNASeq reads from pink and ripening berries. A total of 249,757 and 233,087 putative sRNA loci were identified in the primary and secondary assemblies, respectively, and over 80% of these produced 24 nucleotides (nt) sRNAs (Supplementary Fig. 4a, b). Small RNA clusters were filtered if they matched tRNA, rRNA, and snRNA. An additional 30,365 clusters in the primary assembly and 27,822 clusters in the secondary assembly produced RNA products outside the range of 20–24 nt and were also removed. The remaining clusters were classified as small RNA loci and examined for overlap with annotated gene loci and repetitive element loci to assess potential targets of their gene silencing function. In the primary assembly 179,246 out of 216,241 (82.9%), small RNA loci overlapped with repetitive regions, while only 15.3% of the loci were located in gene bodies including exons and introns (Supplementary Fig. 4a and Supplementary Table 4). Similar small RNA loci distribution was seen in the secondary assembly (Supplementary Fig. 4b and Supplementary Table 4). The small RNA density on the chromosomes was computed using the same approach as genes and repeats and revealed that a majority of small RNA loci colocalized with annotated repeats (Fig. 1b and Supplementary Fig. 4).

We identified 638 putative microRNA loci, which are important for RNA silencing and post-transcriptional gene regulation. Of these, 552 microRNA loci are conserved with 315 microRNAs from 86 plant species recorded in miRBase, and the remaining 86 are novel microRNAs specific to *V. darrowii* (Supplementary Table 5). Based on normalized expression values, the most abundant microRNA in all four libraries is homologous to Arabidopsis miR165. Other abundant microRNAs during berry development in blueberry included miR162, miR7725, miR159, miR1167, miR5181, miR3633, miR6248, and miR397. A total of 1102 microRNA and mRNA pairs from the primary genome were predicted via psRNATarget^28^ However, future work to verify the computationally predicted gene targets is needed to better integrate the miRNA and gene regulatory networks controlling berry development.

### Genes associated with berry development

**Fig. 2:**
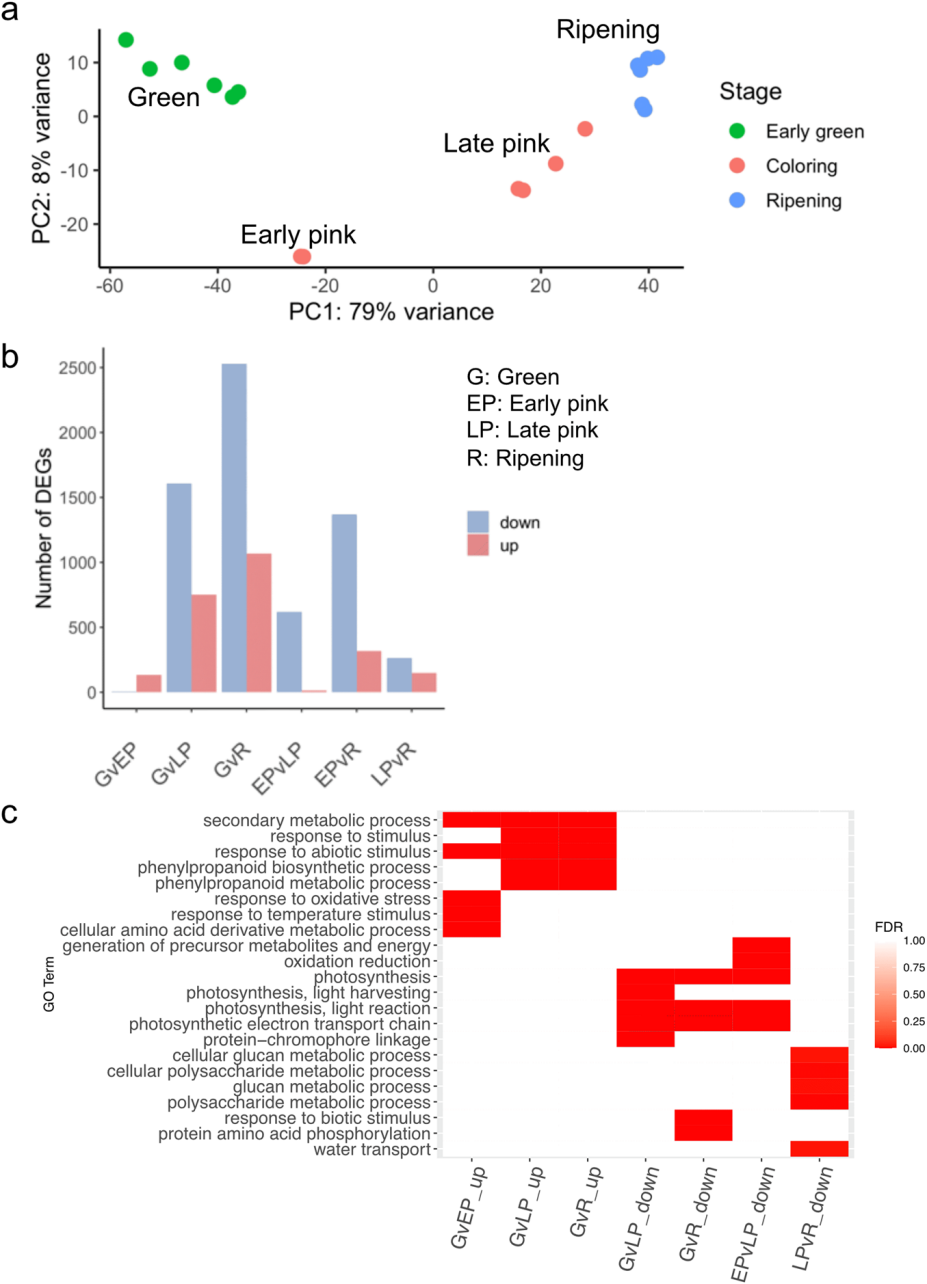
Transcriptome changes during *V. darrowii* berry development. **a** Principal component analysis showed transcriptome variance in samples by developmental stage. The largest principal component encompasses 79% of the variation (horizontal axis) while the second principal component spans only 8% of the variation. **b** The number of differentially expressed genes in the pairwise comparison among four berry stages. **c** Gene ontology enrichment of the top five GO terms enriched in the upregulation or downregulation DEGs

We collected *V. darrowii* fruits in the stages of early (green), coloring (pink), and ripening (blue) and performed RNA sequencing to quantify gene expression. An average of 90.34% of the reads uniquely mapped to the primary genome assembly (Supplementary Table 7). Out of 34,809 annotated genes, 27,595 were found to be expressed in at least one sample and their expression profiles were subjected to principal component analysis (PCA). Distinctive expression profiles were identified between berries in green and ripening stages, with 79% variance along the first principal component (Fig. 2a). Berry samples in the coloring stage were clustered into two groups, in which two samples closer to the green stage were defined as ‘early pink’ and four samples with small progress to the ripening stage were defined as ‘late pink’ (Fig. 2a). Differential expression analysis was performed between each pair of stages. Consistent with the PCA, we identified the largest number of differentially expressed genes (DEGs) between green and ripening stages (GvR). The low number of DEGs in green vs. early pink (GvEP) also indicated a small variance of expression profiles between these two stages (Fig. 2b). In all the comparisons except for GvEP, more downregulated genes were identified than upregulated genes.

To broadly understand the biological functions of DEGs in berry development, we performed GO enrichment analysis on the upregulated genes and downregulated genes separately. The top five enriched GO terms from biological processes were visualized in Fig. 2c. The upregulated genes in the pink and ripening stages were involved in the secondary metabolic process (GO:0019748, FDR < 2.79e−5), phenylpropanoid biosynthesis (GO:0009699, FDR < 8.5e−9) and phenylpropanoid metabolic process (GO:0009698, FDR = 8.5e−9), and cellular amino acid derivative metabolic process (GO:0006575, FDR = 4.3e−5). The downregulated genes in the late pink and ripening stages were mainly involved in the photosynthesis pathways including photosynthesis (GO:0015979, FDR < 1.6e−17), photosynthesis, light reaction (GO:0019684, FDR < 8.7e−9), photosynthetic electron transport chain (GO:0009767, FDR < 2.5e−6) and photosynthesis, light-harvesting (GO:0009765, FDR = 1.3e−5). Genes associated with response to temperature stimulus (GO:0009266, FDR = 4.0e−6) and response to oxidative stress (GO:0006979, FDR = 3.3e−10) were only upregulated in the early pink stage but oxidation reduction-related genes were downregulated in the ripening stage. Surprisingly, genes involved in the cellular polysaccharide metabolic process (GO:0044264, FDR = 6.1e−3) and cellular glucan metabolic process (GO:0006073, FDR = 0.015) were downregulated between late pink and ripening stages (Fig. 2c). Our results suggest that berries undergo different biological processes in different developmental stages. Green berries initially have some photosynthetic processes that are then reduced in the coloring and ripening stages. Genes involved in abiotic stresses and biosynthesis of antioxidant compounds were significantly upregulated during the progression of fruit development.

**Fig. 3:**
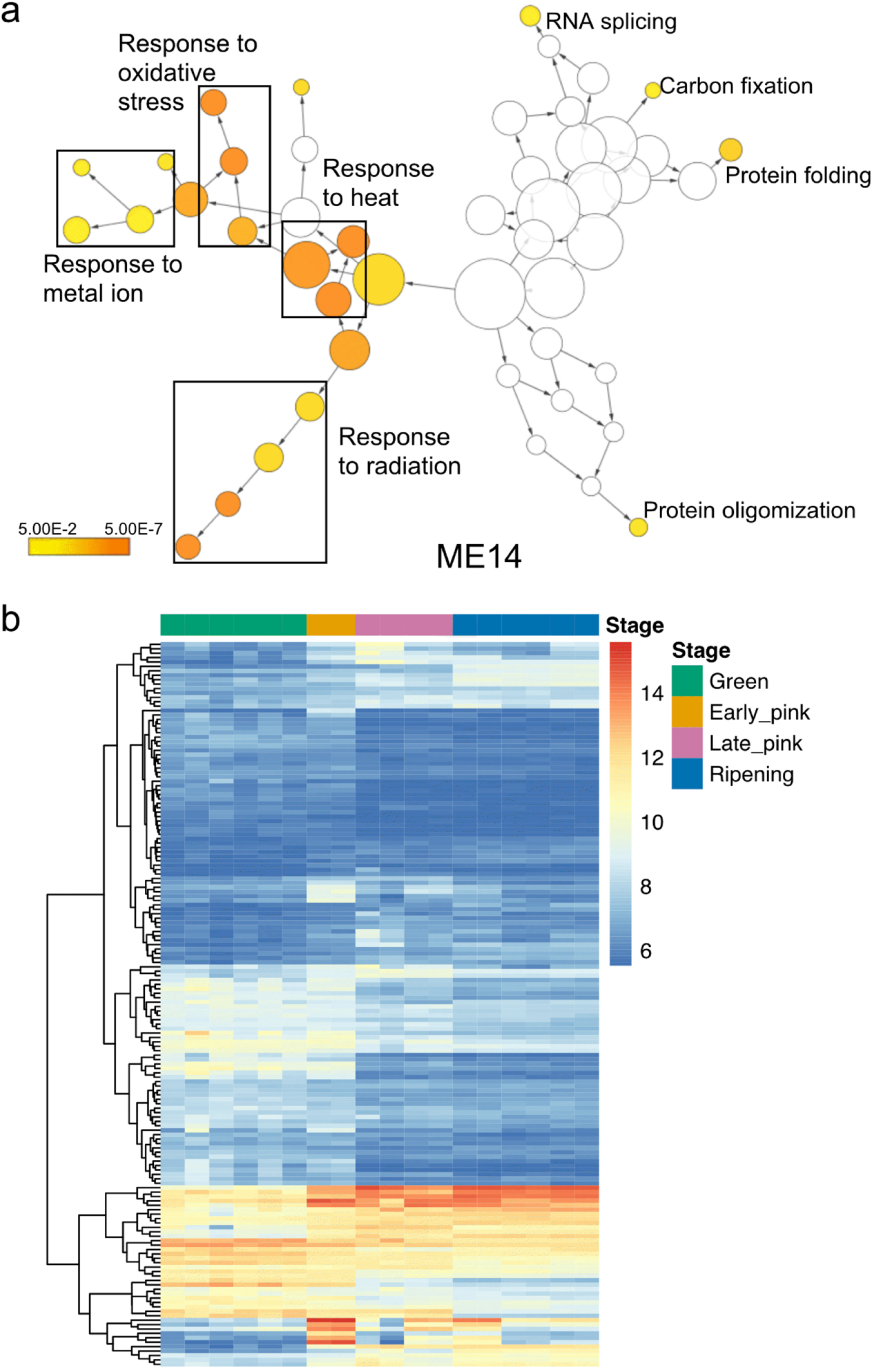
Functional enrichment. **a** GO enrichment networks of differential expression genes in ME14, the module with the strongest correlation to the green berry stage. **b** Gene heatmap of the expression of DEGs involved in oxidative stress

We then identified genes that shared common expression patterns over fruit development stages by co-expression analysis. The 27,595 expressed genes were clustered into 15 modules (ME0 to ME14), which contained varying numbers of member genes from 432 (ME14) to 10,532 (ME1). Module-factor’s relationship of gene modules and berry stages were calculated by Pearson correlation coefficient (PCC) with positive values indicating high expression level in that stage, and negative values representing low expression levels in that stage. We selected four modules with the highest PCC values at each stage: ME14 with the highest correlation to the green stage, ME11 with the highest correlation value to the early pink stage, ME7 with the highest correlation to the late pink stage, and ME9 with the highest correlation to the ripening stage (Supplementary Fig. 5a, b). GO enrichment analysis for the DEGs from ME14 (green berry stage) identified significant associations with response to oxidative stress, response to metal ions, and response to heat and radiation (Fig. 3a). Highly expressed genes from ME11 (early pink) were enriched in calcium ion transport (Supplementary Fig. 6). Calcium is important for fruit size and prevents blueberry fruit drop during the early stages of fruit development^29^. Calcium uptake in berries was found to accelerate in the early green stage, then reduce during the coloring stage and cease completely in the ripening stage^30^. Consistent with these findings, our gene expression profiles showed high expression levels of genes associated with calcium transport and calcium ion response in the green and early pink stages.

### Expression profiles of flavonoid and photosynthesis genes

**Fig. 4:**
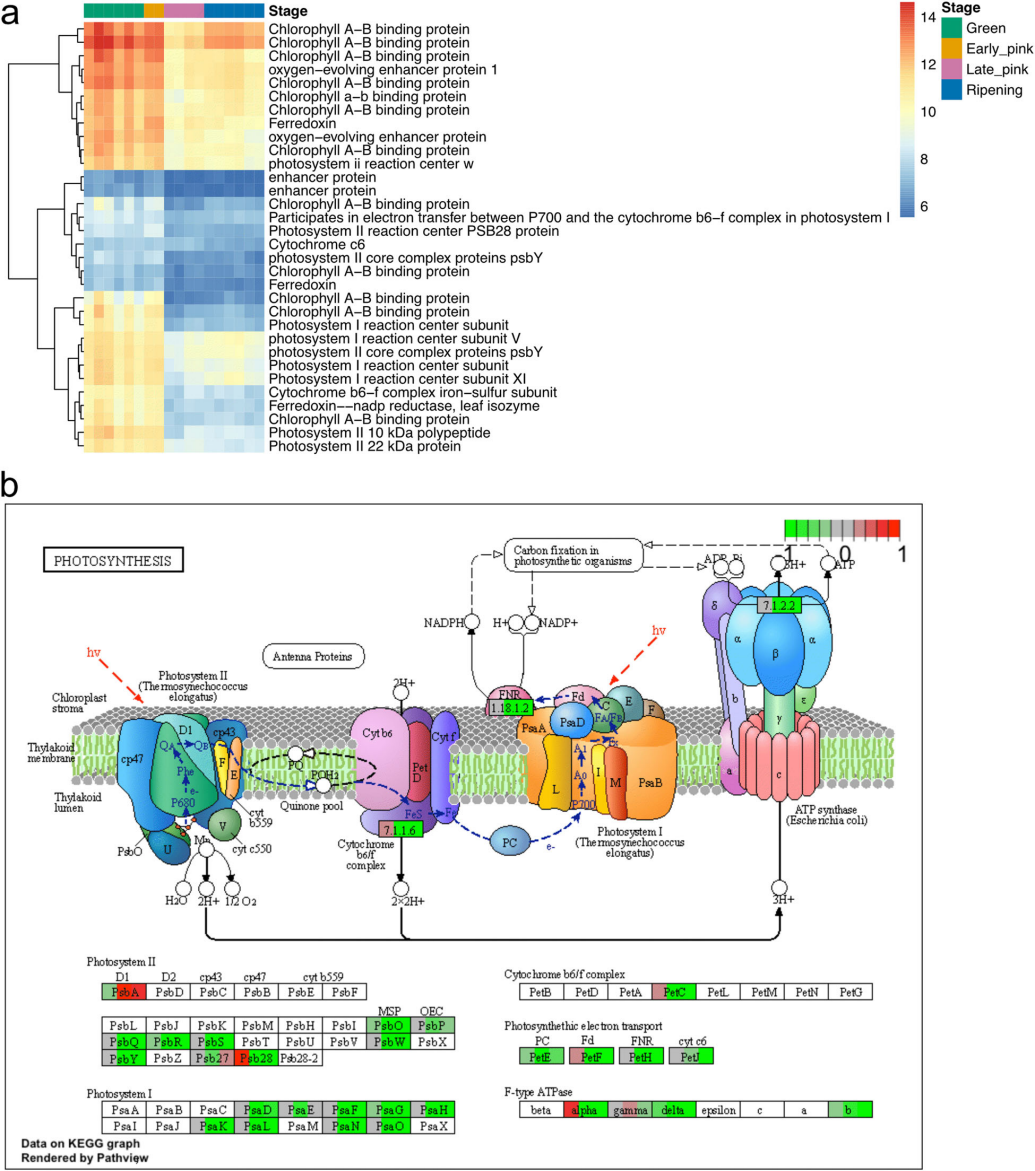
Expression profile of DEGs involved in photosynthesis. **a** Heatmap of photosynthesis gene expression profiles of differentially expressed genes from at least one stage. **b** The KEGG pathway diagram of photosynthesis overlaid with *V. darrowii* DEG expression changes. The expression pattern for each gene is visualized as colors in the boxes containing the gene name. Each box is divided into three colored sections, corresponding to the log2 fold change in early pink, late pink, and ripening stages compared to the green stage

Based on the DEG and co-expression network results, the overall patterns of gene expression for photosynthesis pathways and flavonoid and anthocyanin pathways were further annotated and investigated. Photosynthesis was depleted in the late stages of fruit development (Fig. 2c). We identified 66 genes in the photosynthesis pathway (ko 000195 and ko 000196), and 32 of these genes were differentially expressed in at least one of the comparisons. The DEGs in the photosynthesis pathway were expressed in high levels at the green and early pink stages and were downregulated in the late pink and ripening stages (Fig. 4a). Gene expression changes were visualized and integrated into the KEGG pathway for photosynthesis (accession ko00195). The key genes in photosystem I and II were all downregulated in the late pink and ripening stages, except for PsbA (Fig. 4b). PsbA protects photosystem II proteins from oxidative damages in maize^31^. The upregulation of PsbA in the late stages of berry development suggests that oxidative stress might increase during berry development.

**Fig. 5:**
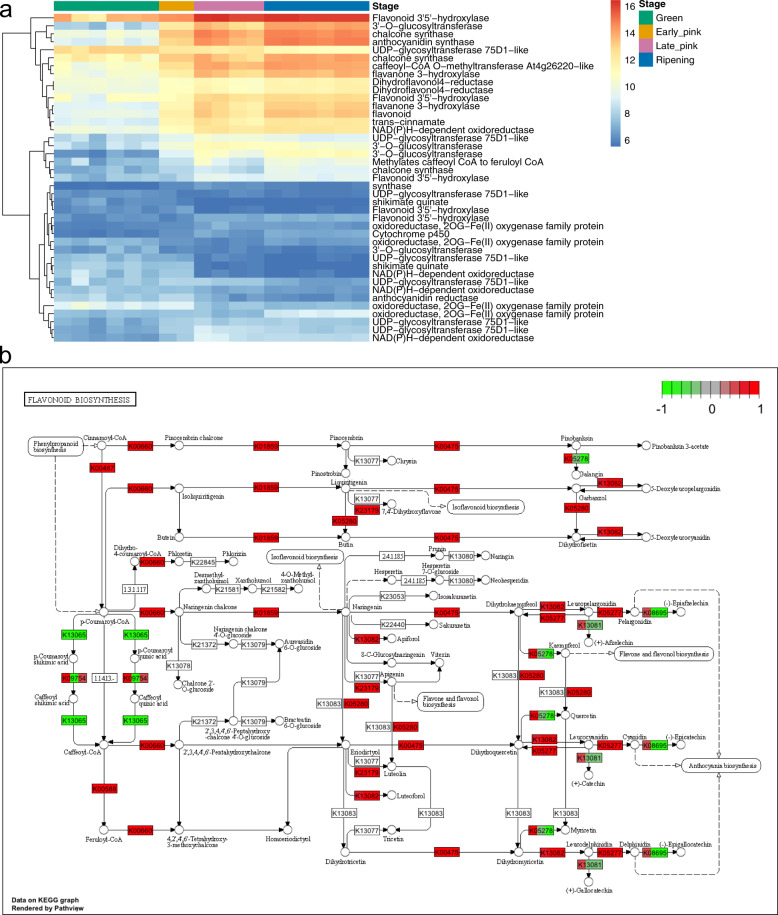
Expression profiles of DEGs involved in flavonoid biosynthesis pathways. **a** Heatmap of flavonoid biosynthesis gene expression profiles of differentially expressed genes from at least one stage. **b** The KEGG pathway diagram of flavonoid biosynthesis pathways overlaid with *V. darrowii* DEG expression changes. The expression pattern for each gene is visualized as colors in the boxes containing the gene name. Each box is divided into three colored sections, corresponding to the log2 fold change in early pink, late pink, and ripening stages compared to the green stage

We then investigated the flavonoid and anthocyanin biosynthesis pathways genes. There are 129 genes annotated in the flavonoid biosynthesis pathway (ko00941) and anthocyanin biosynthesis pathway (ko00942). Forty-one out of 129 genes were identified as DEGs and their overall expression pattern indicated increases in expression over the stages of berry development with 28 genes upregulated and 13 genes downregulated as berry development proceeded (Fig. 5a). Genes in the flavonoid biosynthesis pathway had increased expression levels from the early pink stage to the late pink and ripening stages (Fig. 5b). The gene encoding anthocyanidin reductase (*ANR*), known to catalyze pelargonidin, cyanidin, and delphinidin to epiafzelechin, epicatechin, and epigallocatechin, was significantly decreased (Fig. 5b). The bronze1 (*BZ1*) gene, encoding an anthocyanidin 3-O-glucosyltransferase responsible for anthocyanin accumulation, was highly expressed during berry ripening (Supplementary Fig. 6). Our results on the key genes involved in flavonoid and anthocyanin biosynthesis pathways suggested that the intermediate secondary metabolites in the anthocyanin pathways may start to accumulate at the early stage of fruit development.

### Comparison of anthocyanin related genes between *V. darrowii* and *V. corymbosum*

As *V. darrowii* has a history of being crossed to *V. corymbosum*, identifying orthologs between the species and species-specific genes could be of interest for breeding programs in order to understand the beneficial contributions to cultivated SHB from *V. darrowii*. We identified 18,907 homologous genes between *V. corymbosum* and *V. darrowii* by reciprocal best hits from BLAST results (Supplementary Fig. 7). To find orthogroups across all haplotypes, analysis was expanded to include 193,085 genes representing two haplotypes in *V. darrowii* and four haplotypes in *V. corymbosum* using OrthoFinder^32^, which resulted in 156,724 genes assigned to 37,767 orthogroups. The orthogroups included 62,240 (96.5%) genes from *V. darrowii* and 94,484 (73.5%) genes from *V. corymbosum* (Supplementary Figure 9). Out of 37,767, 25,401 orthogroups included at least one gene from both species (Supplementary Fig. 9). To evaluate if the genes identified in *V. darrowii* but not *V. corymbosum* were truly unique, a BLAST analysis of the *V. darrowii* specific genes against the *V. corymbosum* primary haplotype genome was conducted. Results found 3652 of 4502 genes had alignments of more than 200 bases and identity of more than 70%, potentially indicating these genes are present but not annotated in *V. corymbosum*. 1815 genes aligned to intergenic regions and were enriched in DNA integration (FDR = 1.44e−51), RNA-dependent DNA replication (FDR = 2.20e−42), and DNA metabolic process (FDR = 1.18e−40). These genes are likely associated with repetitive elements that were masked before annotation. The remaining 850 genes with no clear ortholog in *V. corymbosum* may be unique to *V. darrowii* and are candidates for follow up study to understand the unique trait contributions of *V. darrowii* to breeding programs (Supplementary Table 8). These genes have enriched functions for transport (GO:0006810, FDR = 8.39E−05) and reproductive processes (GO:0022414, FDR = 6.39E−06).

**Fig. 6:**
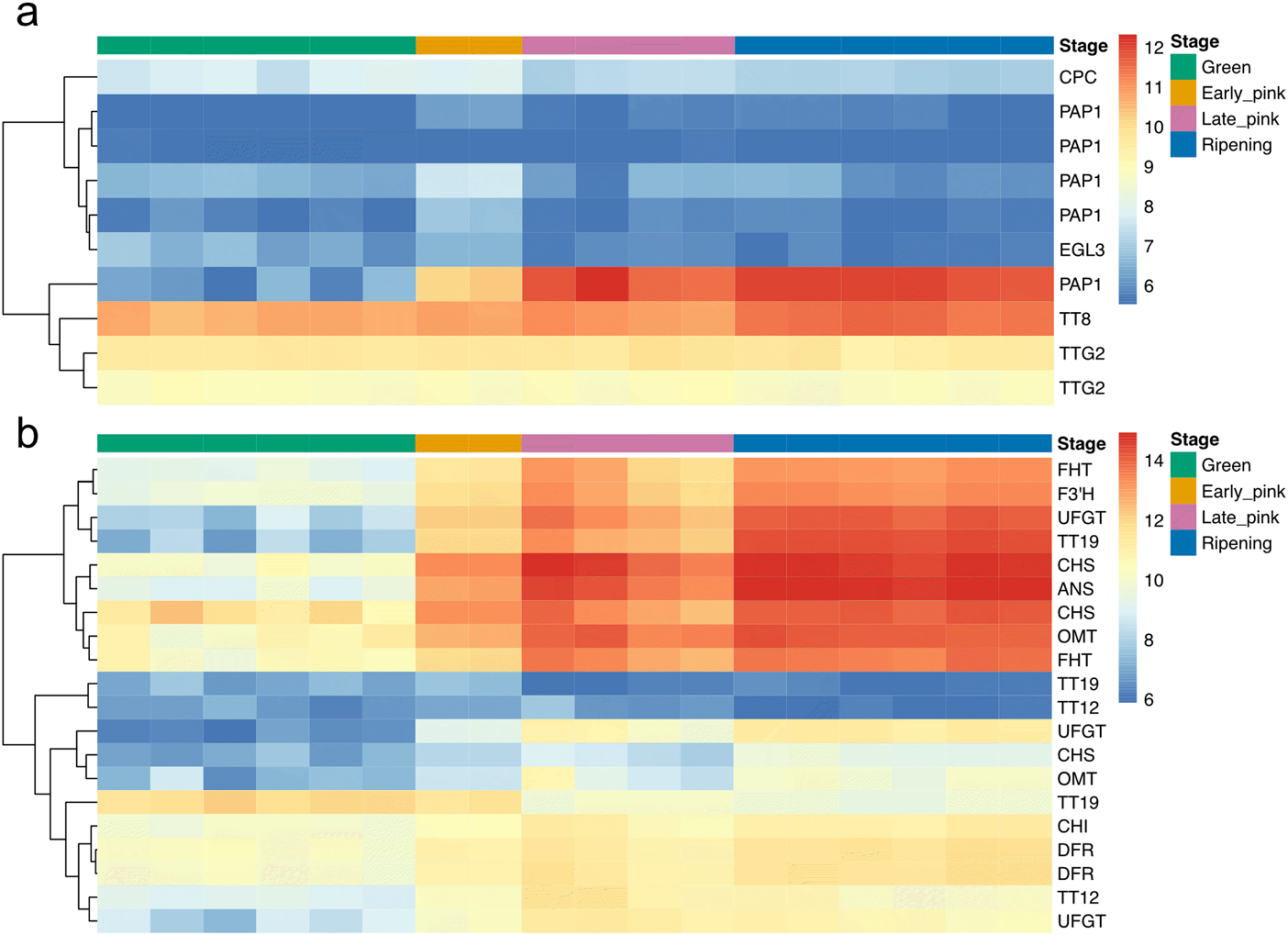
Expression profiles of genes homologous to *V. corymbosum* anthocyanin biosynthesis related genes. Anthocyanin biosynthesis-related transcription factors (**a**) and genes (**b**) homologous to *V.corymbosum*

Colle et al.^15^ reported 90 genes involved in anthocyanin biosynthesis and 35 transcription factors regulating anthocyanin biosynthesis from the four haplotypes of the ‘Draper’ genome. In the *V. darrowii* primary genome, 10 transcription factors and 20 additional genes are homologous to those identified in the ‘Draper’ anthocyanin biosynthetic pathway (Supplementary Table 9, Fig. 6b, c). Two transcription factors, PAP1 (VaDar_g20442) and TT8 (VaDar_g42349) showed increased expression levels during berry development, especially in ripe berries, while other transcription factors did not have significant changes (Fig. 6a). Of five PAP1 homologs, two (VaDar_g20437 and VaDar_g20442) were found to be significantly upregulated at the early pink stage (Supplementary Table 9). The majority of anthocyanin biosynthetic genes increased in expression from the green berry stage to the ripe berry stage (Fig. 6b). Consistent with *V. corymbosum*, genes *ANS*, *UFGT*, and *OMT* involved in producing anthocyanin began upregulation at the coloring stages and the expression levels continued to increase throughout the fruit development stages (Fig. 6b).

## Discussion

Blueberry is a commercially important fruit crop in the United States. It originated in North America and is currently grown worldwide^33^. Genomic resources are expanding with a recently published reference genome for the NHB tetraploid species *V. corymbosum*^15^. The tendency of certain clones of *V. darrowii* to produce unreduced gametes has been used successfully to reduce the chilling requirement and introduce traits from several *Vaccinium* species into highbush blueberries. As a ‘bridging species’ providing genetic resources to generate SHB, a reference genome for *V. darrowii* is essential to identify genomic variations and potential molecular markers between NHB and SHB cultivars. A recent population genetics study of SHB and NHB with SNP markers was not able to genetically distinguish the two groups, highlighting the need for genomic resources and more in-depth research to distinguish DNA segments carrying important traits^19^.

Across many crop species, surprisingly extensive genomic structural variation has been found within populations, spurring new interest in accurately profiling this type of variation and moving from a single individual reference genome paradigm to more comprehensive pangenomes^34^^,^^35^. Further, structural variation has been found to drive traits of interest in fruit and grain crops^36–40^. Here, we present a diploid, chromosome-level genome for *V. darrowii* with two haplotype-level assemblies. The primary assembly is the most complete, with more bases (582.7 Mb vs. 480.5 Mb) and a higher number of genes (34,809 vs. 29,717) than the secondary assembly (Table 1). The availability of draft genomes of both haplotypes represents an important resource for structural heterozygosity and allelic variation present within *V. darrowii*. Using the haplotype assemblies and long reads for *V. darrowii*, we found over 360 well-supported structural variants present in this individual, indicating structural variation is a promising target for future efforts to understand the genomic drivers of phenotypes of interest in blueberry breeding. However, with the combination of PacBio long reads and HiC data, current algorithms are not capable of resolving a fully phased haplotype-resolved assembly without parental or progeny information^41,42^. New technology such as 10x Genomics linked-read sequencing and sequencing of progeny in a breeding program will be needed to define and track haplotype blocks.

Synteny analysis of *V. corymbosum* and *V. darrowii* showed high collinearity between the two genomes (Fig. 1a). Chromosome translocations among the four haplotypes in the ‘Draper’ genome suggest that tetraploid *V. corymbosum* is an autopolyploid derived from a highly diverse species^15^. Such chromosome-level translocations were not found between the two haplotypes of the *V. darrowii* genome (Fig. 1b). However, eight intrachromosomal rearrangements were observed when comparing the primary haplotype assemblies of *V. corymbosum* to *V. darrowii* (Fig. 1 and Supplementary Fig. 1). Alignment and examination of PacBio long reads confirmed that the *V. darrowii* gene order in the primary assembly was correct, suggesting the rearrangements between *V. darrowii* and *V. corymbosum* are real and not an assembly artifact.

Many plant genomes consist of a high number of repetitive sequences, largely composed of transposable elements. Transposable element content estimates from the two previously published Vaccinium reference genomes largely agree 39.53% of the American cranberry (*V. macrocarpon*) genome^43^ and 44.35% of the tetraploid ‘Draper’ NHB genome^15^. In *V. darrowii*, we identified a total of 492,735,410 bases (46.34% of the diploid genome) as repetitive sequences (Supplementary Table 3). Across all three species, similar distributions of the repetitive classes were also observed. LTR elements including *Copia* and *Gypsy* were the most abundant repetitive elements in all four sequenced genomes, and long interspersed nuclear elements were the main proportion of non-LTR elements (Supplementary Table 3)^44^.

Small RNA sequencing identified 552 conserved microRNAs and 86 novel predicted microRNAs in *V. darrowii* berries, which are more than the number of microRNAs previously identified in *Vaccinium ashei*^45^. This may be due to the higher depth of sequencing in this study, which may have captured additional lowly expressed miRNAs. The two most abundant microRNAs (chr1_1990384 and chr10_2208602), located on chromosome 1 and chromosome 10, were characterized as homologs of Arabidopsis miR165a (Supplementary Table 5). MiR165 is known as an abundant microRNA class in plants and is involved in multiple plant developmental processes including shoot apical meristem differentiation, root stele differentiation, and regulation of positional specialties of leaf tissues^46^. The mature miR165 is identical to miR166 except for one nucleotide difference at the seventeenth position. MiR165 and miR166 have the same gene targets and are reported to be involved in abscisic acid (ABA) and auxin responses in abiotic stresses^47,48^. Current computational tools to predict microRNA targets generate many false-positive results that require intensive validation including degradome sequencing or rapid amplification of 5’ complementary DNA ends (5′RACE) PCR. Further investigations will be needed with more biological replicates coupled with degradome sequencing to identify the specific targets of these microRNAs during berry development.

Fruit ripening is associated with significant changes in the expression of genes associated with sugar accumulation, cell wall degradation, and anthocyanin accumulation. In this study, RNA-Seq analysis revealed changes related to photosynthesis pathways and flavonoid and anthocyanin pathways. Gupta et al.^18^ reported that more than half of the genes involved in the photosynthesis pathway were significantly downregulated in pink and ripe berries in SHB “O’Neal”. Consistent with that study in *V. corymbosum*, *V. darrowii* genes in the photosynthesis pathway were expressed in high levels at the green and early pink stages and were downregulated in the late pink and ripening stages (Fig. 4A). Downregulation of genes in the photosynthesis pathway corresponds to chlorophyll degradation occurring during late pink and ripening stages. Cyanogenic glycoside biosynthesis genes were also found to be highly expressed in the green stage of berry development in *V. corymbosum*, likely as a defense compound against herbivores consuming unripe berries with immature seeds. Nitrilase 4 (*NIT4*) encodes an enzyme that degrades cyanogenic glycoside into asparagine. It was upregulated in the ripe berries, which suggested that the detoxification of cyanogenic glycoside likely occurred during berry ripening^18^. We found that genes involved in the cyanogenic glycoside biosynthesis pathway and *NIT4* in *V. darrowii* showed similar expression profiles as *V. corymbosum* (Supplementary Fig. 10). Our results suggest that expression patterns of genes associated with defense and antioxidants during berry development are conserved between *V. corymbosum* and *V. darrowii*.

*V. darrowii* has been used as a source of superior heat and drought tolerance for blueberry breeding^49^. The efficiency of breeding blueberries with both heat tolerance and high fruit quality could be accelerated by identifying genes associated with these traits and developing molecular markers for marker-assisted breeding. In green berries of *V. darrowii*, GO enrichment analysis identified highly expressed genes associated with response to heat and radiation (Supplementary Fig. 6 and Fig. 4a) which are not reported in SHB or northern highbush blueberry^15,18^. Results from this study detected attractive candidate genes and provided a good platform for further functional characterization of genes associated with heat tolerance and anthocyanin accumulation in blueberry.

## Materials and methods

### Plant materials

The diploid blueberry *V. darrowii* clone NJ8810/NJ8807 from the USDA ARS Southern Horticultural Research Laboratory in Poplarville, MS (30.8402° N, 89.5342° W) in this study. For whole-genome sequencing, young leaves of clone NJ8810/NJ8807 were subjected to DNA extraction and sequencing. For transcriptome sequencing, berries from green, coloring, and ripening stages were subjected to RNA extraction and RNASeq, with details below in DNA and RNA sequencing.

### DNA and RNA sequencing

Young leaves were collected from a single plant of *V. darrowii* clone NJ8810/NJ8807 and used for genomic DNA extraction using a modified hexadecyltrimethylammonium bromide (CTAB) protocol^19^. Isolated DNA was quantified with a Nanodrop 2000 spectrophotometer and a Qubit dsDNA HS assay kit (ThermoFisher Scientific, Waltham, MA). DNA quality was assessed using an Agilent 2100 Bioanalyzer (Agilent Tech, Santa Clara, CA, USA). The TruSeq Nano DNA Libraries were constructed using Truseq Nano DNA sample preparation kit (Illumina, San Diego, CA, USA) following the manufacturer’s protocol. DNA libraries were used in paired-end sequencing (2 × 150 bp, 30× coverage) on the Illumina HiSeq 3000 sequencer at the USDA ARS Genomics and Bioinformatics Research Unit Stoneville, MS.

Young expanding leaves, dark treated for 48 h prior to harvest, were collected from *V. darrowii* clone NJ8810/NJ8807 in liquid nitrogen and used to isolate high molecular weight (HMW) DNA. HMW DNA was used to construct SMRTbell^TM^ libraries following the manufacturer’s protocol and sequenced using the Pacific Biosciences (PacBio) sequencing platform (Pacific Bioscience, CA, USA). Hi-C library preparation and sequencing were performed by Dovetail Genomics using their standard methods (Santa Cruz, CA, USA).

For RNA isolation six biological replicates from each of green berries (green), pink berries (pink), and ripe berries (blue) were collected from *V. darrowii* (Supplementary Table 10). The green berries were collected when the majority of berries were green, the pink stage was collected when 50% or more of the berries were pink, and the ripe berries were collected when 50% of the berries were fully ripe. RNA was extracted using the Spectrum^TM^ Plant Total RNA Kit (Sigma-Aldrich) as described by the manufacturer. The quantity of the isolated RNA was examined using a Nanodrop 2000 spectrophotometer and a Qubit 2.0 Fluorometer using the RNA broad range kit (Life Technologies, Carlsbad, CA, USA). RNA integrity was evaluated using Agilent 2100 Bioanalyzer (Agilent Tech, Santa Clara, CA, USA), and samples with integrity scores greater than 8.5 were used for library preparation. Libraries were constructed using the NEBNext^TM^ II Directional RNA library prep kit for Illumina (NEB, MA, USA). Libraries with an insert size of about 350 bp were sequenced using NovaSeq 6000. Three 20 kb SMRTbell template libraries prepared from three RNA samples were sequenced on a PacBio Sequel instrument following the PacBio Iso-Seq protocol.

### Genome assembly

*De novo* assembly was performed using MECAT2^50^. The draft genome assembly was polished by consensus calling with Arrow from PacBio SMRT Tools using the PacBio long reads. The genome assembly was further polished by two rounds of error correction with Pilon using Illumina short reads^51^. At this stage the assembly represented a partially fused set of heterozygous sequences representing both haplotypes. This mostly diploid representation of the genome was separated into a primary haplotype assembly and secondary haplotype assembly by purge_dups^20^. To anchor contigs into chromosomes, the primary assemblies were scaffolded using Hi-C sequencing reads through the Dovetail HiRise^TM^ pipeline (Dovetail Genomics, LLC). The secondary haplotype assembly was scaffolded using the same Hi-C data with the software packages juicer and 3D-DNA^21,22^.

### Genome annotation

The primary assembly and secondary haplotype assembly (i.e., the haplotigs removed by purging) were annotated separately using RNAseq, Iso-Seq, and *V. corymbosum* proteins^15^ by the BRAKER2 pipeline^52^. First, repetitive elements were identified and masked by RepeatModeler^24^ and RepeatMasker^53^ using previously characterized plant repetitive elements from RepBase^54^. Next, trimmed Illumina RNASeq reads were aligned to both masked assemblies by STAR v2.7.3a^55^. Iso-Seq reads were aligned to genome assemblies by minimap2^56^. Both aligned RNASeq and Iso-Seq reads were merged by samtools^57^ and used as transcript evidence in the BRAKER2 pipeline. Additionally, 128,559 proteins from the *V. corymbosum* ‘Draper’ genome^15^ were mapped to masked assemblies by Prothint^58^ as protein evidence. Repeat masked assemblies were annotated by BRAKER2 under ‘etpmode’ with both transcript and protein evidence. The predicted gene models were then filtered with structural and functional annotation by EnTAP and gFACS^59,60^. Functional annotation including GO terms, KEGG entries, and gene functions for the gene models were assigned by EnTAP searching against databases including RefSeq^25^, Uniprot^26^, and eggNog^27^. Genome completeness was assessed by BUSCO v4.0 on the genome and gene models using the embryophyta lineage^61^. Genome continuity was evaluated using LAI in the LTR_retriver package^23^.

### sRNA sequencing and analysis

Four of the RNA samples from the berry tissues were also subjected to small RNA sequencing. A total of 52,528,351 adapters removed small RNASeq reads were obtained from a colored berry sample and three ripening berry samples. Small RNASeq reads were first aligned to large and small subunits of ribosomal RNA (rRNA) from SILVA to remove potential rRNA contamination^62^. Clean reads were aligned to the primary and secondary assemblies by ShortStack and small RNA loci identified^63^. For each sRNA locus, the major sRNA sequence was defined using the most abundant sRNA sequence. The major RNA sequence for each small RNA cluster was compared to the Rfam database with BLAST using an *e*-value cutoff of 0.1 to further filter tRNA, rRNA, and snoRNAs. Small RNA clusters overlapping with gene bodies and repetitive elements were identified by ‘bedtools intersect’ function^64^.

MicroRNA loci on the primary assembly were identified by miRDeep2^65^ using mature and precursor microRNA sequences from 86 plant species in miRBase^66^. MicroRNA structures were predicted by RNAfold in the ViennaRNA package 2.0^67^. MicroRNA loci with a miRDeep2 score greater than 5 and a randfold *p*-value greater than 0.05 were kept. These predicted microRNAs were then quantified by ‘quantifier.pl’ from miRDeep2 and their expression values normalized through the DESeq2 pipeline^68^. The mRNA targets of microRNAs were predicted by psRNATarget^28^.

### Genome synteny analysis

The primary haplotype assembly was aligned to the secondary haplotype assembly with minimap2 v2.17 in asm5 mode^56^. The alignments were visualized as a dotplot with D-GENIES^69^. To define structural variants, the PacBio reads were mapped to the primary haplotype with minimap2 v2.17 in map-pb mode. Samtools v1.9 was used to convert the output to bam^57^, which was provided to Sniffles v1.0.12a with default parameters^70^. The resulting vcf file was filtered by bcftools v1.9^71^ to retain only variants of at least 10 kb with a minimum depth of 30 PacBio reads supporting the reference haplotype and a minimum depth of 30 PacBio reads supporting the alternate haplotype. A subset of variants was visualized in IGV to confirm the accuracy of breakpoint locations^72^.

The scaffolded primary assembly was compared to a haplotype representation of *V. corymbosum* ‘Draper’ genome^15^ by CoGe Synmap with default parameters^73^. The chromosomes of the primary assembly were renamed to follow the numbering of their corresponding *V. corymbosum* chromosomes. The secondary haplotype assembly was then aligned to the chromosome-ordered primary assembly. Alignment results from DAGChainer were extracted by a customized python script and visualized by Circos^74^ into a circular format and ‘RIdeogram’^75^ in a linear format.

### Gene orthology analysis

To identify the homologous genes between *V. corymbosum* and *V. darrowii*, 128,559 proteins from the tetraploid ‘Draper’ genome and 64,526 proteins from the diploid *V. darrowii* were analyzed by OrthoFinder^32^. Reciprocal best hits between *V. corymbosum* haplotype and *V. darrowii* primary assembly were obtained from NCBI BLAST results with evalue cutoff of 1e−10 and keeping only the first matched hit.

### Gene expression analysis

RNASeq reads were first trimmed by Skewer to remove adapters and low-quality reads^76^. After data quality assessment by FastQC^77^, clean reads were then aligned to *V. darrowii* primary assembly by STAR v2.7.3a^55^. Gene expression was quantified by HTSeq^78^ from uniquely mapped reads and differential expression analysis was performed using the R package ‘DESeq2’^68^. PCA was performed using sample variances calculated by regularized log-transformed gene counts provided by DESeq2. The stages of berry development from colored berries were redefined as ‘early pink’ and ‘late pink’ according to the PCA. A Wald test was used to perform a pair-wise comparison between the berry stages to identify significant DEGs with a fold change greater than 2 and an adjusted *p*-value less than 0.05.

A co-expression network was constructed using Weighted Gene Correlation Network Analysis (WGCNA) based on the normalized gene counts from DESeq2, following the tutorials^79,80^. Genes with no mapped reads across all samples were removed from the analysis. 27,595 genes were subjected to WGCNA to construct 15 co-expression modules (ME0–14). The relationship between modules and berry stages was calculated by PCC.

### GO enrichment analysis

The functional enrichment analysis of significant DEGs or genes from selected co-expression modules was performed by BiNGO in Cytoscape^81^ using the *V. darrowii* primary genome annotation as background. The enriched GO terms were determined by a hypergeometric test following Benjamini and Hochberg’s false discovery rate (FDR) correction. Significant enrichment was identified with FDR less than 0.05. The GO networks were built and analyzed in Cytoscape^82^.

### Identification of KEGG orthologs and visualization of KEGG map

KEGG orthologs were assigned to gene models annotated in the primary assembly using KEGG Automatic Annotation Server^83^. Gene fold changes at each berry stage were obtained by comparing the gene expression levels to the green berry stage using the ‘DESeq2’ R package described above in the differential expression analysis. Gene expression log2 fold changes were visualized in the selected KEGG pathways including photosynthesis (ko00195), flavonoid biosynthesis (ko00941), and anthocyanin biosynthesis (ko00942) from the KEGG database by R package ‘Pathview’^84^.

## Supplementary information

Supplementary Figures

Supplementary Tables

## Data Availability

Illumina RNASeq and Iso-seq reads were deposited to NCBI under BioProject accession PRJNA706655. This whole-genome Sequence including the primary and secondary assemblies have been deposited at DDBJ/ENA/GenBank under the BioProject accession PRJNA706655 and PRJNA707651 with accessions JAFMTH000000000 and JAFMTI000000000, respectively. The python and R scripts used in this study were deposited in https://github.com/statonlab/blueberry-berry-development.
