# Supplementary material for: High-quality reference genome and annotation aids understanding of berry development for evergreen blueberry (Vaccinium darrowii)
Source: Hortic Res. 2021 Nov 1;8:228. doi: 10.1038/s41438-021-00641-9 (PMC8558335; doi:10.1038/s41438-021-00641-9)
Supplement: Supplementary file 1 — Supplementary Figures [file 41438_2021_641_MOESM1_ESM.docx]

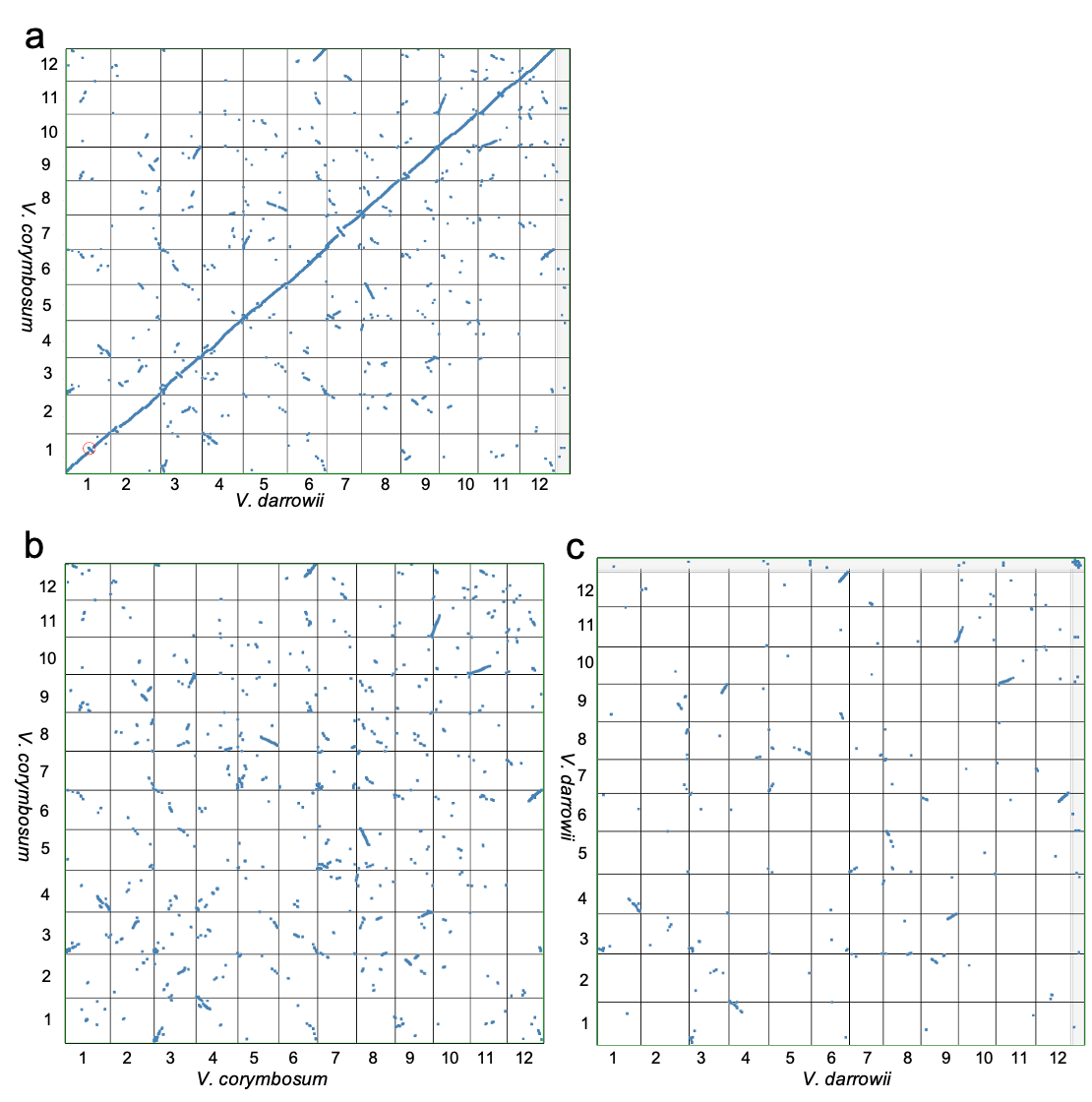


Supplementary Figure 1. Synteny analysis between *V. darrowii* and *V. corymbosum* by SynMap. a. *V. darrowii* to *V. corymbosum* (<https://genomevolution.org/coge/SynMap.pl?dsgid1=59918;dsgid2=60396>). b. *V. corymbosum* to *V. corymbosum* (<https://genomevolution.org/coge/SynMap.pl?dsgid1=60309;dsgid2=60396>). c. *V. darrowii* to *V. darrowii* (<https://genomevolution.org/coge/SynMap.pl?dsgid1=59918;dsgid2=59918>).


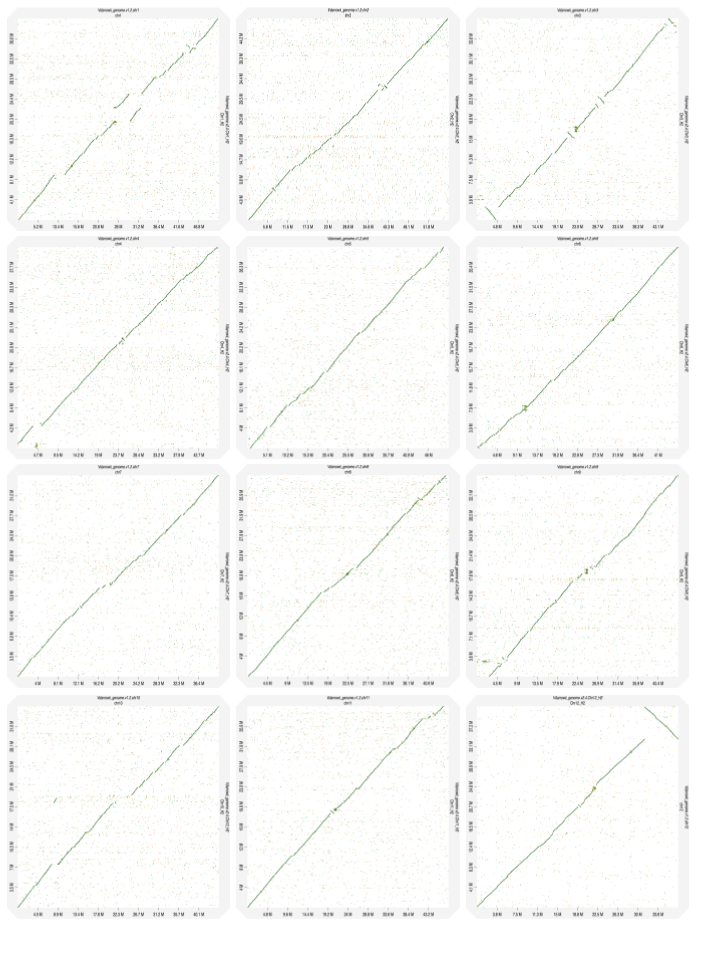


Supplementary Figure 2. Synteny analysis between *V. darrowii* primary and secondary haplotypes by chromosome (http://dgenies.toulouse.inra.fr/result/Vd_Hap1-vs-Hap2-try2)


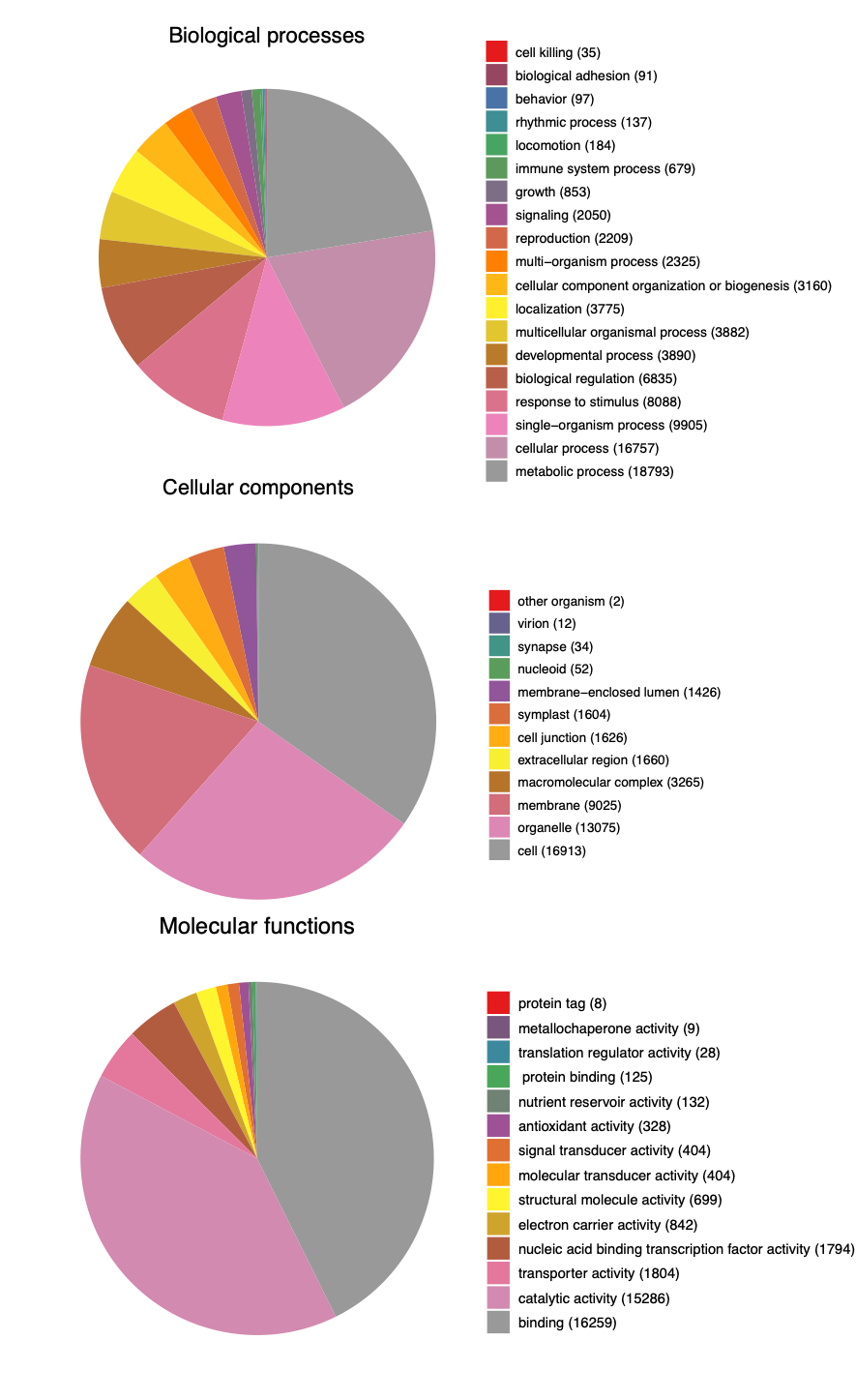


Supplementary Figure 3. Gene ontology annotation of protein-coding genes in the primary assembly.


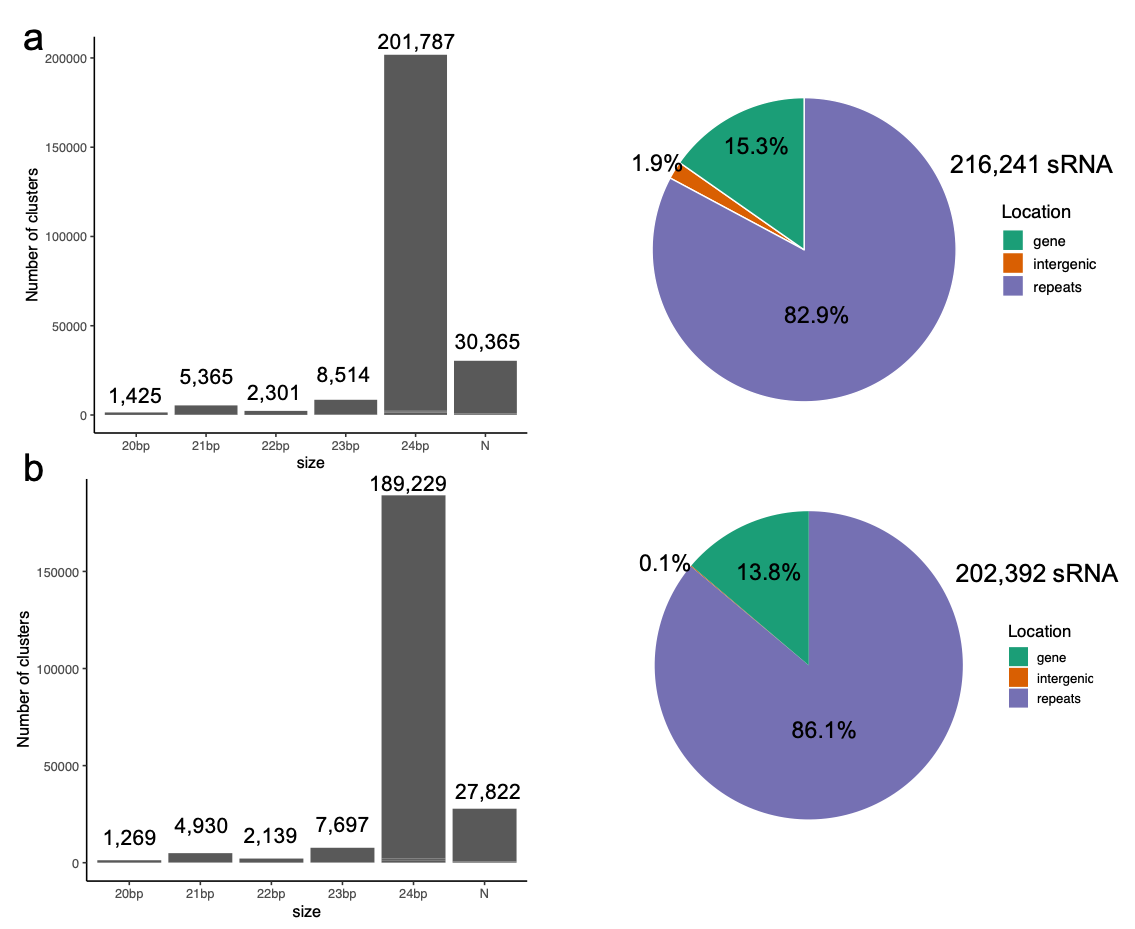


Supplementary Figure 4. (a) small RNA clusters length distribution in the primary assembly (b) small RNA clusters length distribution in the secondary assembly.


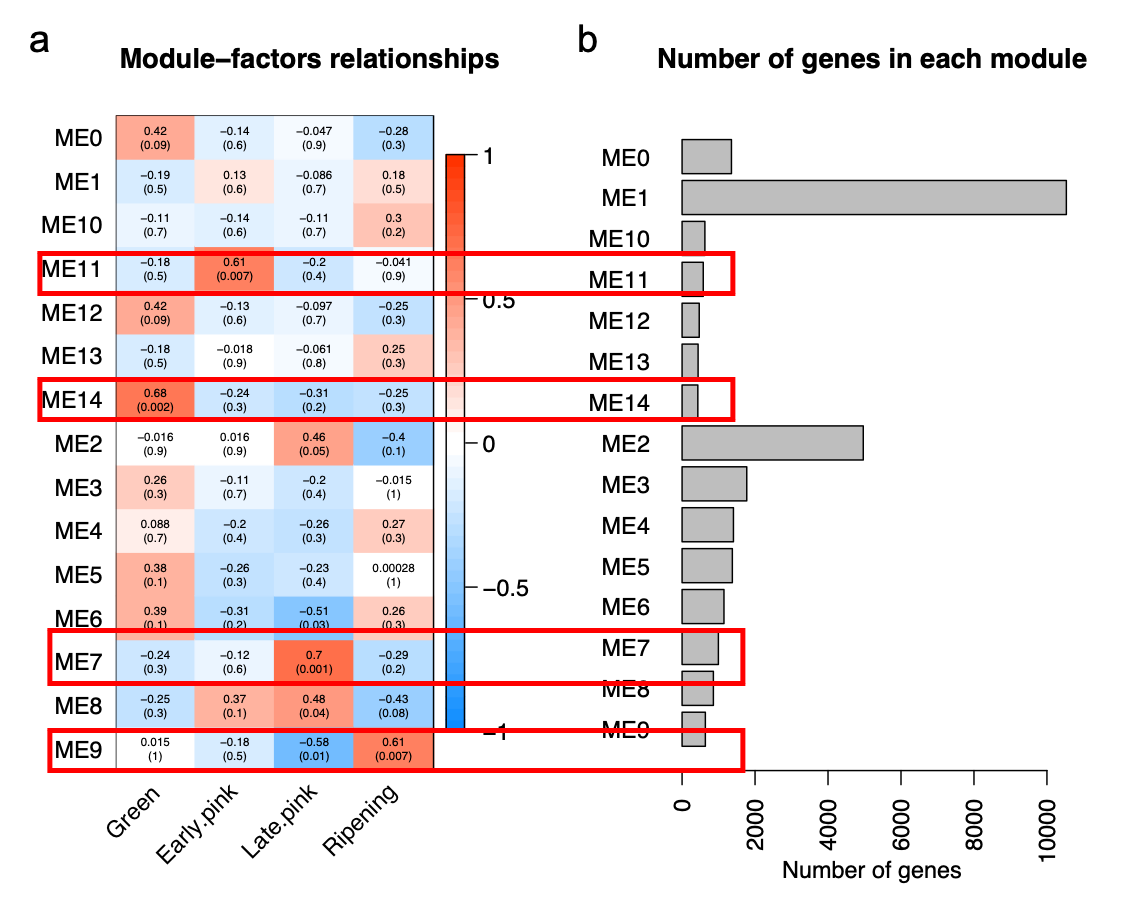


Supplementary Figure 5. Co-expression modules and the number of genes in each module. (a) Module-factor relationships heatmap. Higher correlation values indicated higher expression level at the stage. (b) The number of genes included in each ME.


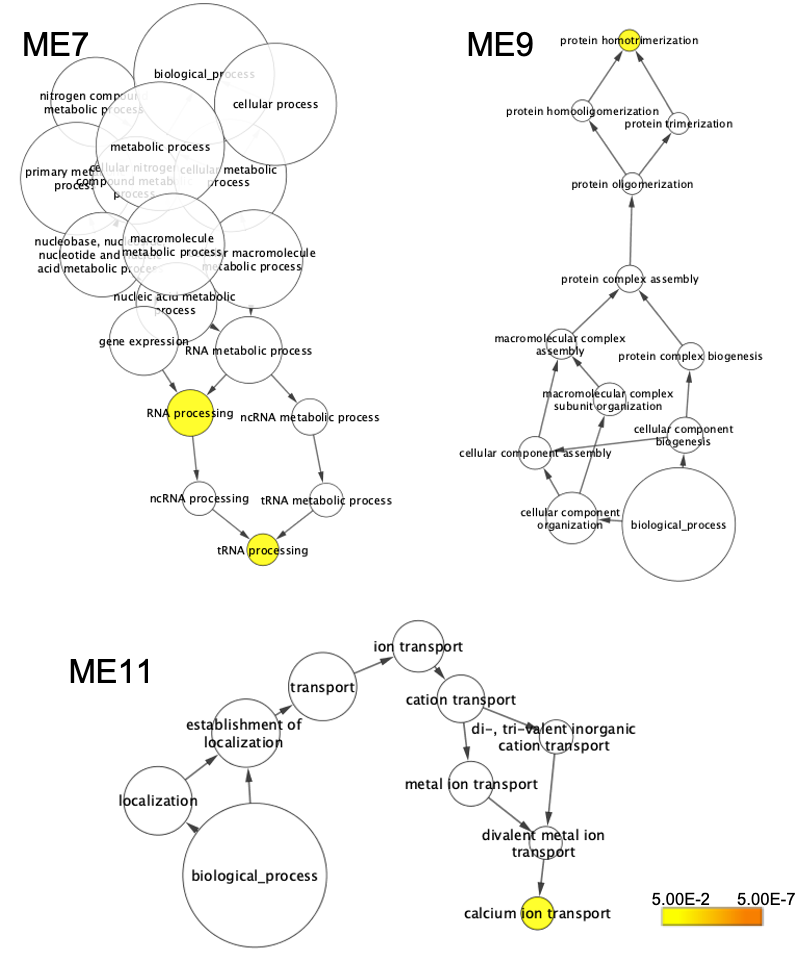


Supplementary Figure 6. GO enrichment networks of DEGs from ME7, ME9 and ME11.


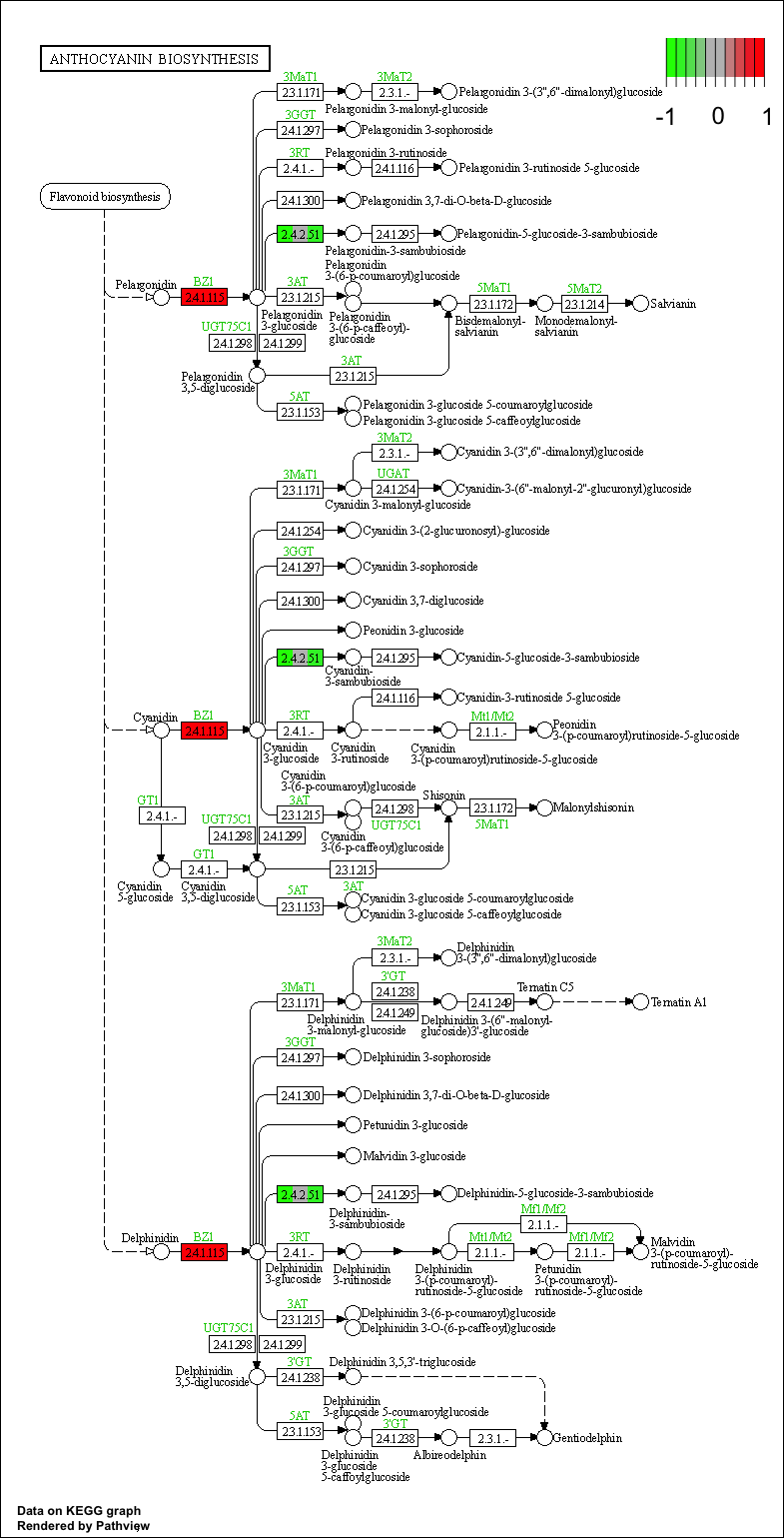


Supplementary Figure 7. KEGG pathway diagram of anthocyanin biosynthesis pathway (ko00942). The color scale in the pathway map showed the log2 fold change in pink and ripening stages compared to the green stage.


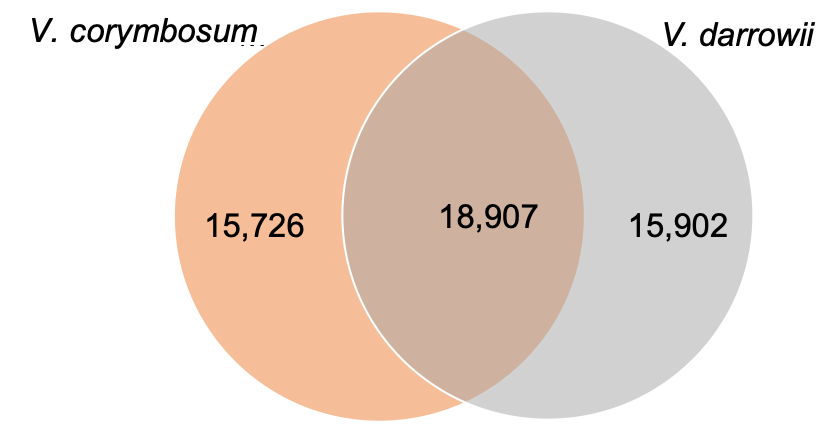


Supplementary Figure 8. The number of genes homologous with high confidence identified by reciprocal best hit between *V. corymbosum* and *V. darrowii*.


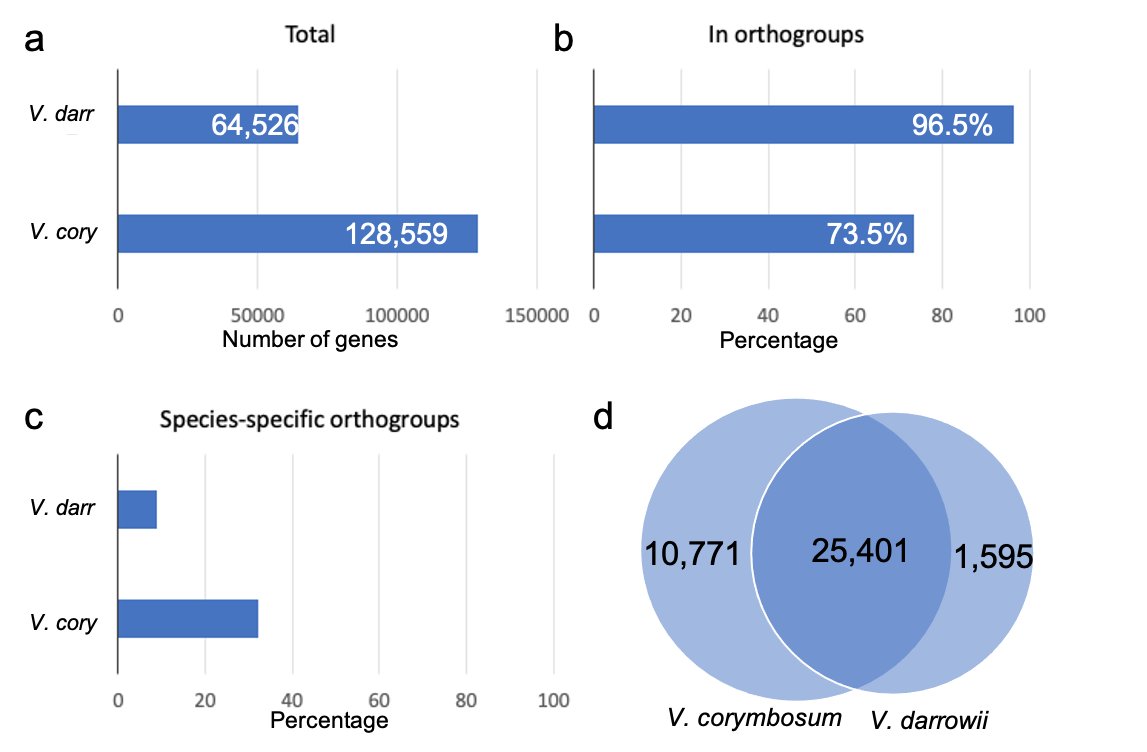


Supplementary Figure 9. Orthogroups between *V. corymbosum* and *V. darrowii.* (a) The number of genes in diploid *V. darrowii* and tetraploid *V. corymbosum.* (b) The percentage of genes grouped into orthogoups. (c) The percentage of orthologs specific to *V. corymbosum* or *V. darrowii.* (d) Overlapping of orthogoups between *V. corymbosum* and *V. darrowii.*

Supplementary Figure 10. Gene expression profiles of cyanogenic glycoside biosynthesis pathway related genes and nitrilase 4 (*NIT4*).
